# The impacts of biomineralization and oil contamination on the compressive strength of waste plastic-filled mortar

**DOI:** 10.1038/s41598-022-25951-3

**Published:** 2022-12-13

**Authors:** Kylee Rux, Seth Kane, Michael Espinal, Cecily Ryan, Adrienne Phillips, Chelsea Heveran

**Affiliations:** 1 Center for Biofilm Engineering, Montana State University, Bozeman, MT 59717 USA; 2 Civil and Environmental Engineering Department, Montana State University, Bozeman, MT 59717 USA; 3 Mechanical and Industrial Engineering Department, Montana State University, Bozeman, MT 59717 USA

**Keywords:** Civil engineering, Composites, Biomineralization

## Abstract

Researchers have made headway against challenges of increasing cement infrastructure and low plastic recycling rates by using waste plastic in cementitious materials. Past studies indicate that microbially induced calcium carbonate precipitation (MICP) to coat plastic in calcium carbonate may improve the strength. The objective of this study was to increase the amount of clean and contaminated waste plastic that can be added to mortar and to assess whether MICP treatment enhances the strength. The performance of plastic-filled mortar was investigated at 5%, 10%, and 20% volume replacement for cement. Untreated, clean plastics at a 20% cement replacement produced compressive strengths acceptable for several applications. However, a coating of MICP on clean waste plastic did not improve the strengths. At 10% replacement, both MICP treatment and washing of contaminated plastics recovered compressive strengths by approximately 28%, relative to mortar containing oil-coated plastics. By incorporating greater volumes of waste plastics into mortar, the sustainability of cementitious composites has the potential of being improved by the dual mechanisms of reduced cement production and repurposing plastic waste.

## Introduction

Cement is the second most-consumed resource after water, and its production generates 5–8% of global anthropogenic greenhouse gas emissions^1,2^. The production of clinker, the binder in cement products, produces carbon dioxide as a byproduct in three different stages: (1) calcination of limestone, clay, or sand (2) fuel combustion from manufacturing in a rotary kiln, and (3) emissions from quarrying and transportation of products^2^. By replacing a fraction of the cement paste with waste materials, such as recycled plastic, the overall concrete composite has the potential to be more sustainable.

Between 1950 and 2015, a total of 6300 metric megatons of primary and secondary (recycled) plastic waste was generated^3^. Approximately 9% of this plastic was recycled, 12% was incinerated, and only 10% of recycled plastic has been recycled more than once^3^. If current production and waste management trends continue, by 2050 about 12,000 metric tons of plastic waste will end up in landfills or the environment^3^. An impediment to recycling is that many plastics are coated with food or oily residues, otherwise known as contaminated waste plastics. Plastics that are commonly recycled (e.g., polyethylene terephthalate (PET) or high density polyethylene (HDPE)) are rarely recycled if contaminated because the plastics must be sorted and washed prior to recycling^4,5^. These additional steps are often not economically viable. Additionally, some recycling processes require high operating temperatures as various polymers have different melting temperatures, resulting in higher costs and energy input^4^. The quality of the final product may also be affected during reprocessing leading to fluctuations in the price of recycled materials^5,6^.

Researchers have attempted to mitigate these problems of low recycling rates and greenhouse gas emissions by integrating waste plastics into cement. The addition of waste materials, including plastic, in reinforced cementitious materials have been extensively investigated^2,7–9^. Plastic in the form of fibers at low replacement percentages enhances concrete material properties, including strength^10–14^. However, incorporating chipped waste plastic as a replacement for aggregate decreases the strength of the composite^14–23^. For example, Manjunath et al. found that incorporating PET waste plastic as coarse aggregate decreased the compressive strength as the proportion of plastic increased. At 20% aggregate replacement, the resulting mortar decreased in compressive strength by 47.1%^20^.

While numerous researchers have studied the effects of using waste plastic as aggregate or fiber reinforcement^10–18,24,25^, very few studies have studied the influence of plastic as a partial cement replacement^10,26^. Reducing the amount of cement needed to produce cementitious composites may improve the sustainability of these materials^10,26^. Liu et al. assessed the mechanical properties of concrete using pulverized HDPE waste plastic at 1–7% weight replacement for ordinary Portland cement. At 1%, 3%, and 7% replacement, the concrete cylinders after 28 days of curing exhibited decreases in compressive strength of 2.72%, 2.96%, and 9.98%, respectively^10^. It remains unknown how chipped waste plastic at higher cement replacements impact mortar strength. To combat the loss of strength, calcium carbonate coating on the waste plastic has been investigated as a method to improve mortar strength^26–28^. The addition of calcium-carbonate-coated plastic increases the cement hydration, which may improve bond strength with fibers, ultimately increasing the overall composite strength^27,28^.

One method to attach a calcium carbonate coating to plastic is through microbially induced calcium carbonate precipitation (MICP). MICP uses microorganisms, such as the common soil bacteria *Sporosarcina pasteurii* (*S. pasteurii*), to produce the enzyme urease. The hydrolysis of urea forms hydroxide and carbonic acid. The hydroxide shifts the pH forming bicarbonate and carbonate. The carbonate combined with the calcium has the potential to form calcium carbonate precipitate. The microbes produce the enzyme that catalyzes the chemical reaction to induce calcium carbonate precipitation and may also become encased in the mineral^29–31^. Different polymorphs of calcium carbonate biomineral (e.g., calcite, vaterite, aragonite) produced by MICP have a range of densities, morphologies, nanoscale moduli and hardnesses, and potentially different reactivities in the cementation reactions^32–34^.

Kane et al. evaluated the compressive strength of plastic-reinforced mortar (PRM) containing 5% weight replacement of cement with MICP-treated plastics (PET, polyvinyl chloride (PVC), low density polyethylene (LDPE), PP, polystyrene (PS), or acrylonitrile butadiene styrene (ABS)). While 5% replacement by untreated plastics significantly reduced PRM strength, the same quality replaced by MICP-treated PET, PVC, or mixed type plastics had compressive strengths comparable to plastic-free mortar. These early results were encouraging; however, critical gaps remain regarding the acceptable replacement volumes of waste plastics in cementitious composites.

In this study, we examine the impact of MICP treatment on plastic-filled mortar (P-FM) strength at greater cement replacement volumes of 10% and 20% using four types of post-use plastic waste (HDPE chips, PVC chips, LDPE chips, and LDPE granules). Next, whether MICP treatment could improve the strength of the mortar prepared using oil-contaminated plastics was investigated. It was hypothesized that treating both clean and contaminated plastics with MICP treatment will improve the compressive strength relative to mortar containing untreated plastics. While the hypothesis did not hold, the results of these studies provide valuable information regarding the strength of P-FM containing large replacement volumes of common waste plastics and suitable applications for oil-contaminated P-FM. These studies advance the knowledge about the interaction between plastic type, oil contamination, and MICP treatment on P-FM strength.

## Materials and experimental methods

### Materials

#### Plastic

**Figure 1:**
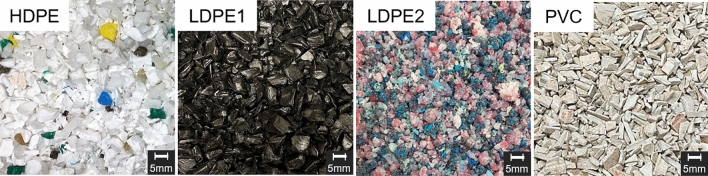
Clean waste plastic gifted by Northwest Polymers.

Post-use, chipped plastic was obtained from Northwest Polymers (Molalla, OR, USA) for addition to P-FM. The clean plastic types included HDPE, PVC, and two different kinds of LDPE (LDPE1 (chips), LDPE2 (granules)). Rubber residue in the PVC was separated from the chipped plastic and discarded. Sieves narrowed the size of the chipped plastic to + 4.75 mm to 9.52 mm. Photos of the waste plastic are shown in Fig. 1.

The density of the chipped plastic was determined using an analytical balance with the density determination kit add-on for solids (Mettler Toledo XS205 Dual Range Balance, Density Kit MS-DNY-54). The density of each plastic type is shown in Table 1.

**Table 1:** Density determined for chipped waste plastic.

Plastic type	Density (g/cm^3^)
HDPE	0.938
PVC	1.441
LDPE1	0.928
LDPE2	0.776

#### Biomineralization solutions

This formerly established MICP protocol has been used in previous studies^26,29^. Solutions used to perform biomineralization are as followed. The calcium-containing solution (CMM+) was composed of 3 g/L nutrient broth, 10 g/L ammonium chloride, 20 g/L urea, and 48 g/L calcium chloride dihydrate. The solution was brought up to volume, stirred to dissolve the chemicals, and adjusted to pH 6.15 using NaOH or HCl prior to adding the calcium chloride dihydrate. The bacteria culture was grown in brain heart infusion (BHI) (37 g/L BHI, 20 g/L urea; BHI + urea). Both solutions were filtered with a 0.22 µm membrane. All chemicals were obtained from Fisher Scientific.

### Biomineralization of plastic

#### Biomineralization protocol

A flask containing 100 mL of BHI was inoculated with the microbe *S. pasteurii*. The culture was grown for 24 h in an orbital shaker at 30 °C and 150 rpm. Mesh bags (EcoWear-Amazon) containing 121 g of individual plastic types were submerged in 700 mL of CMM+ in a 1 L beaker (n = 5). The beakers were placed on a stir plate and inoculated with 14 mL of the *S. pasteurii* culture. The beakers were covered loosely with aluminum foil and left to incubate at 135 rpm. After 48 h, the mesh bags were removed from the CMM+. Plastic was removed and air dried on paper towels for 72 h.

#### Biomineral precipitation on plastic

The change in mass was recorded for plastic fibers after biomineralization. Plastic fibers were used rather than chipped plastic to control for geometry between plastic types. Plastic filament with a diameter of 1.75 mm was purchased from https://Filaments.ca (Mississauga, ON, Canada). Plastic types included PET (PETG Filament—White), HDPE (Filament—Natural), PVC (Filamentum Vinyl 303 PVC—Black), and LDPE (LLDPE Filament—Natural). Filament was cut to a goal length of 8 mm. Fibers were biomineralized as described in “Biomineralization of plastic” section. The mass of the fibers was recorded following biomineralization and air drying for 72 h.

#### Biomineral precipitation on oil-contaminated plastic

The change in mass was recorded with the addition of vegetable oil to plastic fibers to determine the effect of oil on biomineral deposition. Plastic fibers soaked in Crisco pure vegetable oil (125 mL per each 121 g plastic batch) for 10 min (n = 5). Fibers were manually shaken for 15 s to ensure an even coating of oil on all fibers. Fibers were transferred from the plastic container to a mesh bag and submerged into the mineralization solution (“Biomineralization of plastic” section). The mass of the fibers was recorded following biomineralization and air drying for 72 h.

### Determination of compressive strength for biomineralized plastics at varying replacement percentages

Mortar cylinders were produced with a mix ratio of 0.46: 1.0: 2.0 by weight of tap water, Portland cement (Quickrete, Commercial Grade Type I/II), and sand (ASTM-Type C778 Graded Sand, U.S. Silica Company), respectively, consistent with ASTM C109^35^. Portland cement was substituted by volume with MICP-treated and untreated chipped plastics (HDPE, PVC, LDPE1, LDPE2) at 5%, 10%, and 20% replacement (density determined, where an example mix is presented in Table 2). A consistent volume of plastic was added to the mix by using a volume replacement opposed to weight replacement as the densities of the plastics varied. The chipped plastic was subjected to biomineralization and no biomineralization (n = 5/group). These components were mixed in a rotary planetary mixer following ASTM International C305^36^. An expanded table of the mortar mix design for all volume replacements is shown in Table S2.

**Table 2:** Representative mortar mix design for HDPE plastic at 10% cement replacement. Additional mix ratios are shown in Table S2.

Components	Mass (g) per cm^3^ of mortar	Total (kg) (n = 5)
Cement	19.405	20.29
Sand	9.2	9.47
Water	40	41.19
HDPE plastic	0.595	0.31

The mortar was cast into cylindrical molds (2 in D × 4 in H; Bio-cylinder, Deslauriers Inc.) using the procedure described in ASTM C192^37^. The specimens were cast in two layers. Each layer was compacted with tamping and tapping techniques. The specimens were stored in a concrete curing room at 100% relative humidity for 24 h. After initial curing, the specimens were demolded and cured for an additional 27 days in the curing room.

Compressive strength tests were performed after 28 days for cured mortar cylinders. The specimen height and area were recorded before placing neoprene caps on both sides of the cylinder to ensure an even load rate. The specimens were subjected to compression until failure using a constant load rate of 0.127 mm/s (0.005 in/s) on a MTS Criterion Model 64. A total of five replicates were tested for each plastic type and treatment.

### Mineralogical characterization of biomineral precipitated with and without oil contamination

Biomineral crystalline structures were examined using X-ray diffraction (XRD, Bruker D8 Advance Powder X-ray Diffractometer). Plastic fibers were used to characterize and image the biomineral to control for geometry between plastic types. Mineral was scraped 7 days and 145 ± 5 days off plastic fibers and pulverized using a mortar and pestle to obtain homogeneity. The fine powder was mixed with isopropyl alcohol and dried on a glass slide. Samples were analyzed from 3° to 75° 2θ. Peaks were identified using JADE software. High-resolution images of biomineralized fibers and oil-coated, biomineralized plastic fibers were obtained by a Zeiss Supra 55VP field emission scanning electron microscope (FESEM) with an SE2 detector at 2–3 kV and a working distance of 5.5–8.5 mm. Prior to analysis, fibers were fixed onto carbon fiber tape and sputter-coated with gold (Emitech K-875X Sputter Coater).

### Evaluating the impact of biomineralization versus washing on mortar cylinder strength

Mortar cylinders were prepared to determine whether compressive strength is generated from the biomineral treatment or washing of fibers, that occurs incidentally to biomineralization. Oil-treated fibers were subjected to washing and/or biomineralization. Oil-contaminated HDPE, LDPE1, and LDPE2 were subjected to water or *S. pasteurii* and CMM+ (n = 5/group). Mortar specimens were produced with these fibers at a moderate volume replacement of 10% and tested for compressive strength, as described in “Mineralogical characterization of biomineral precipitated with and without oil contamination” section. All specimens were tested after 75 days of curing.

### Data analysis

All analyses were performed in Minitab (ver. 19.2020.1, Minitab LLC, State College, PA, USA). The criteria for statistical significance was set a priori to 0.05. The effects of plastic type, biomineralization treatment or oil coating, and the interaction between the variables on precipitate mass were examined. One-factor and two-factor ANOVA were used to evaluate the effects of plastic type and oil on the amount of mineral precipitated on the fibers due to biomineralization. Three-factor ANOVA tested the effects of plastic type, biomineralization, and replacement percentage as well as the interaction between these variables on the compressive strength of the mortar. Two-factor ANOVA was also used to evaluate the effects of plastic type, biomineralization treatment or washing, and the interaction between the variables on compressive strength with contaminated waste plastics. In the case of significant interactions, post hoc testing was performed by Tukey multiple pairwise comparisons to distinguish simple effects. All models were checked for residual normality and homoscedasticity.

## Results

### Biomineral can be deposited on clean and oil-coated plastics

**Figure 2:**
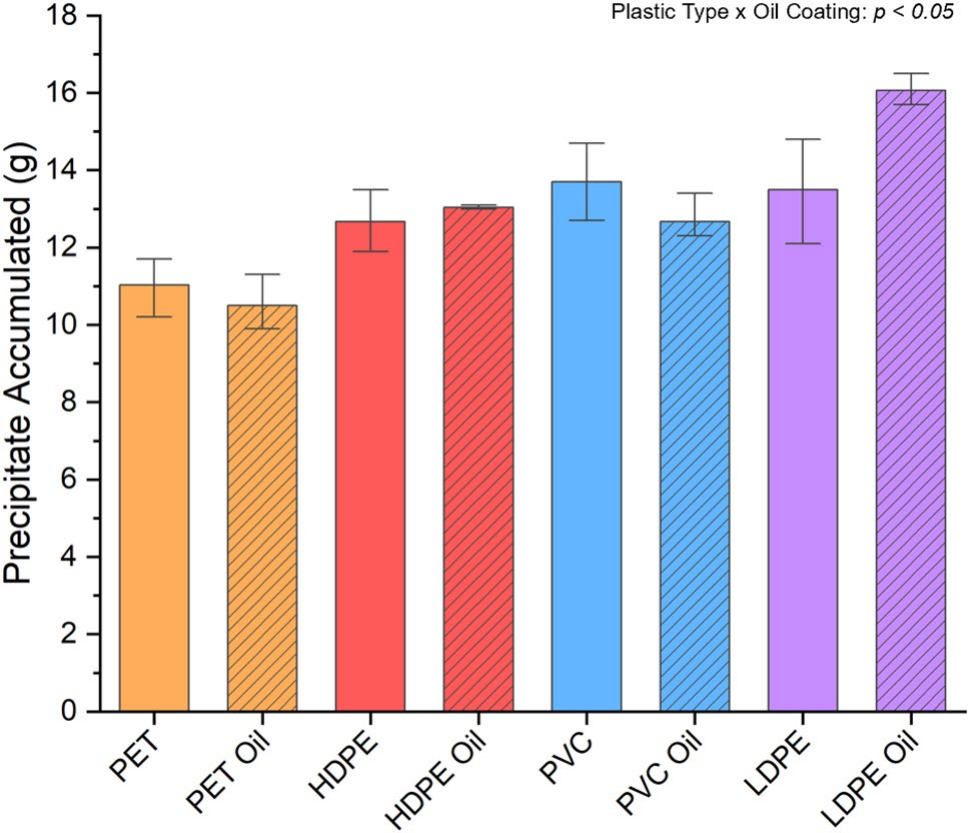
Mass of calcium carbonate attached to 121 g of each plastic type. The bar heights represent the mean, and the error bars indicate one standard deviation.

Calcium carbonate mineral precipitated on all plastic types, regardless of an addition of oil. An increase in mineral on oil-coated LDPE (16.07 ± 0.40 g) was observed relative to clean LDPE (13.5 ± 1.35 g) (Fig. 2). A significant interaction (*p* < 0.05) was found between plastic type and oil coating on the amount of precipitate accumulated. The mean precipitated mineral varied from 9.9 g on oil-coated PET to 16.5 g on oil-coated LDPE. Post hoc Tukey comparisons revealed that oil-coated LDPE exhibited a 19% increase in mineral, relative to clean LDPE (*p* < 0.05). For all other plastic types tested (PET, HDPE, PVC), an oil coating did not impact the amount of mineral precipitated.

### Oil contamination of plastic changes biomineral polymorph and geometry

**Figure 3:**
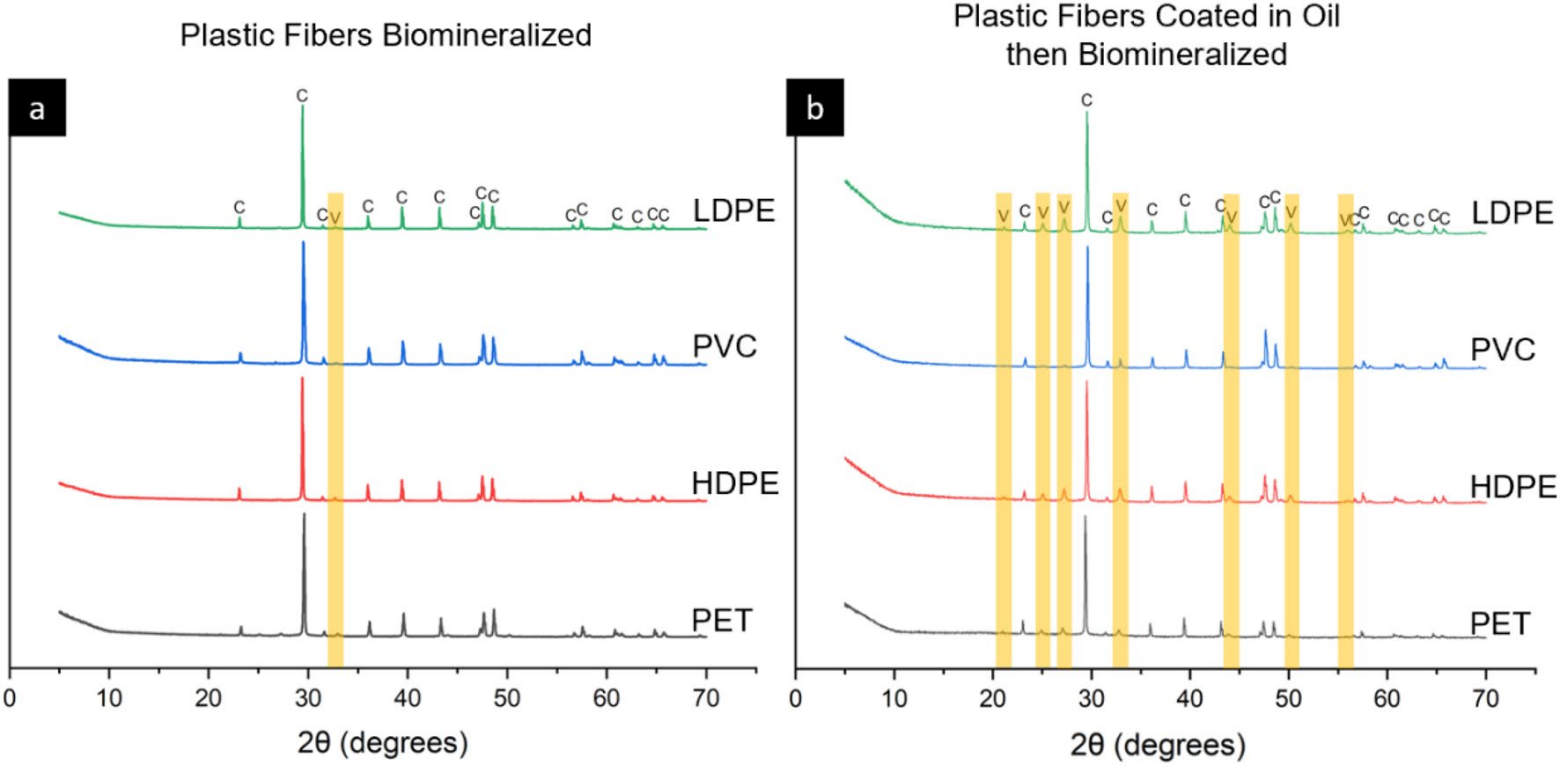
Mineralogy of all samples. (**a**) Mineralized plastic with no oil coating (**b**) Oil-coated and mineralized plastic. Vaterite peaks are highlighted.

XRD shows that MICP produced two calcium carbonate polymorphs (Fig. 3). The predominate mineral on clean plastic fibers was calcite with a minor phase of vaterite. Calcite and vaterite peaks were also observed for oil-coated plastic fibers, although the vaterite signature was larger, suggesting that this phase was more prominent for plastics with oil.

Past work has shown that the metastable vaterite undergoes a phase change to calcite over time^32,38^. Vaterite was still identified on the oil-coated plastics after 145 ± 5 days of aging (Fig. S3). These results possibly indicate that biomineralized oil-coated plastic fibers will have similar properties to freshly biomineralized oil-coated fibers, even after an extended period of time.

**Figure 4:**
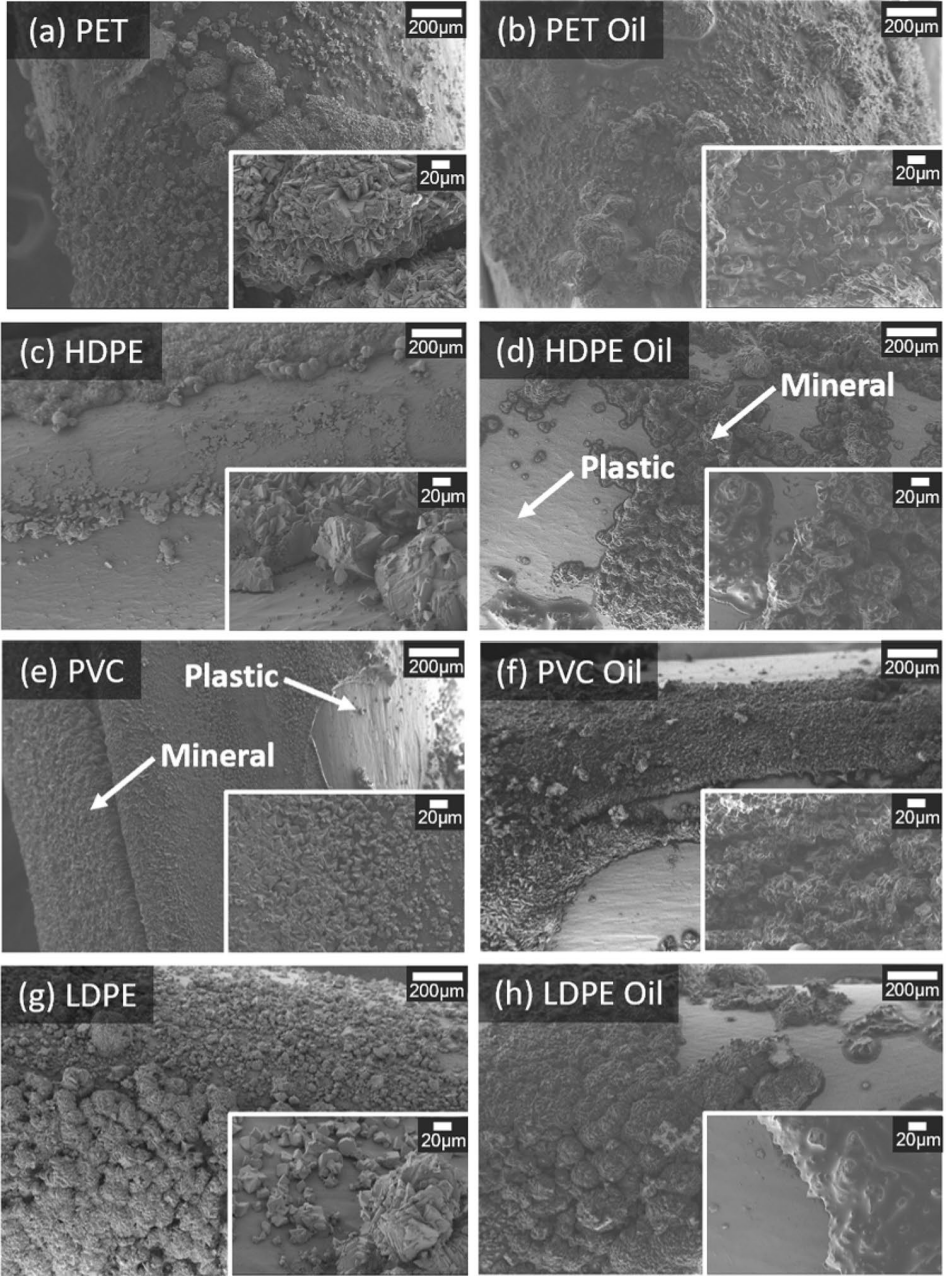
Scanning electron micrographs of biomineralized plastic types with (**a,c,e,g**) and without (**b,d,f,h**) the addition of oil at magnifications of 150x and inset images at magnification of 1000x.

The mineral coverage varied for all plastic types with and without oil (Fig. 4). The calcium carbonate mineral on plastic with no oil coating yielded a rhombohedral morphology (Fig. 4a,c,e,g). This shape corresponds to the mineral calcite, agreeing with the results obtained by the XRD analysis (Fig. 3). Hexagonal and spherical minerals were observed on oil-coated HDPE and PVC (Fig. 4d,f) which exhibited higher vaterite content from XRD analysis (i.e., relatively higher vaterite peaks as compared with the dominant polymorph, calcite) (Fig. 3). The vaterite mineral shape was not distinct on oil-coated plastics PET and LDPE (Fig. 4b,h).

### Plastic type and replacement percentage influences mortar strength

**Figure 5:**
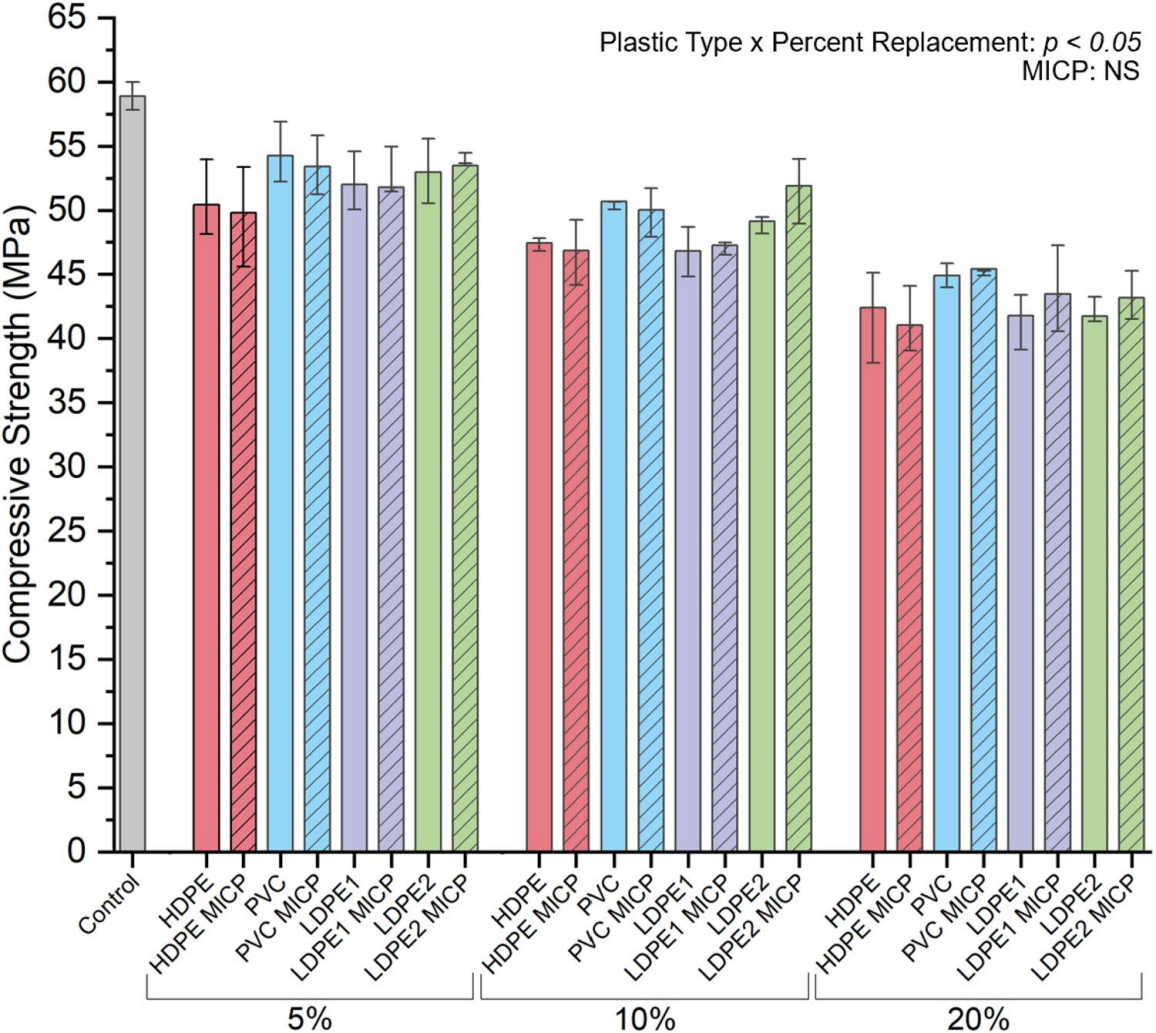
Compressive strengths at replacement percentages 5–20% with waste plastic and no treatment (solid) and MICP-treated waste plastic (striped). Each color indicates a different type of waste plastic: HDPE (red), PVC (blue), LDPE1 (purple), and LDPE2 (green). The bar heights represent the mean, and the error bars indicate one standard deviation.

Larger decreases in mean compressive strength were observed with increasing untreated plastic content. The mean compressive strengths of P-FM prepared with untreated plastic were 11.02% (51.29 MPa), 17.64% (48.50 MPa), and 27.67% (42.60 MPa) less than the mean strength of plastic-free mortar at replacement percentages of 5%, 10%, and 20%, respectively (Fig. 5). The 28-day compressive strength decreased 0.57 MPa for every additional 1% of untreated plastic (R^2^ = 0.70, *p* < 0.05). Subsequently, it was tested whether MICP would improve P-FM strength at each of these replacement quantities.

MICP treatment did not significantly affect the compressive strength of the mortar (*p* > 0.05). However, there was a significant interaction (*p* < 0.05) between the effects of plastic type and the percent plastic replacement on the P-FM compressive strength. Post hoc analysis showed that cylinders with 10% PVC and 10% LDPE2 both exhibited significantly higher compressive strengths than either HDPE or LDPE1 at a 10% replacement. Mortar cylinders containing PVC at a 20% replacement had significantly higher compressive strength than any of the other plastic types at this replacement percentage (*p* < 0.05) (Fig. 5).

**Figure 6:**
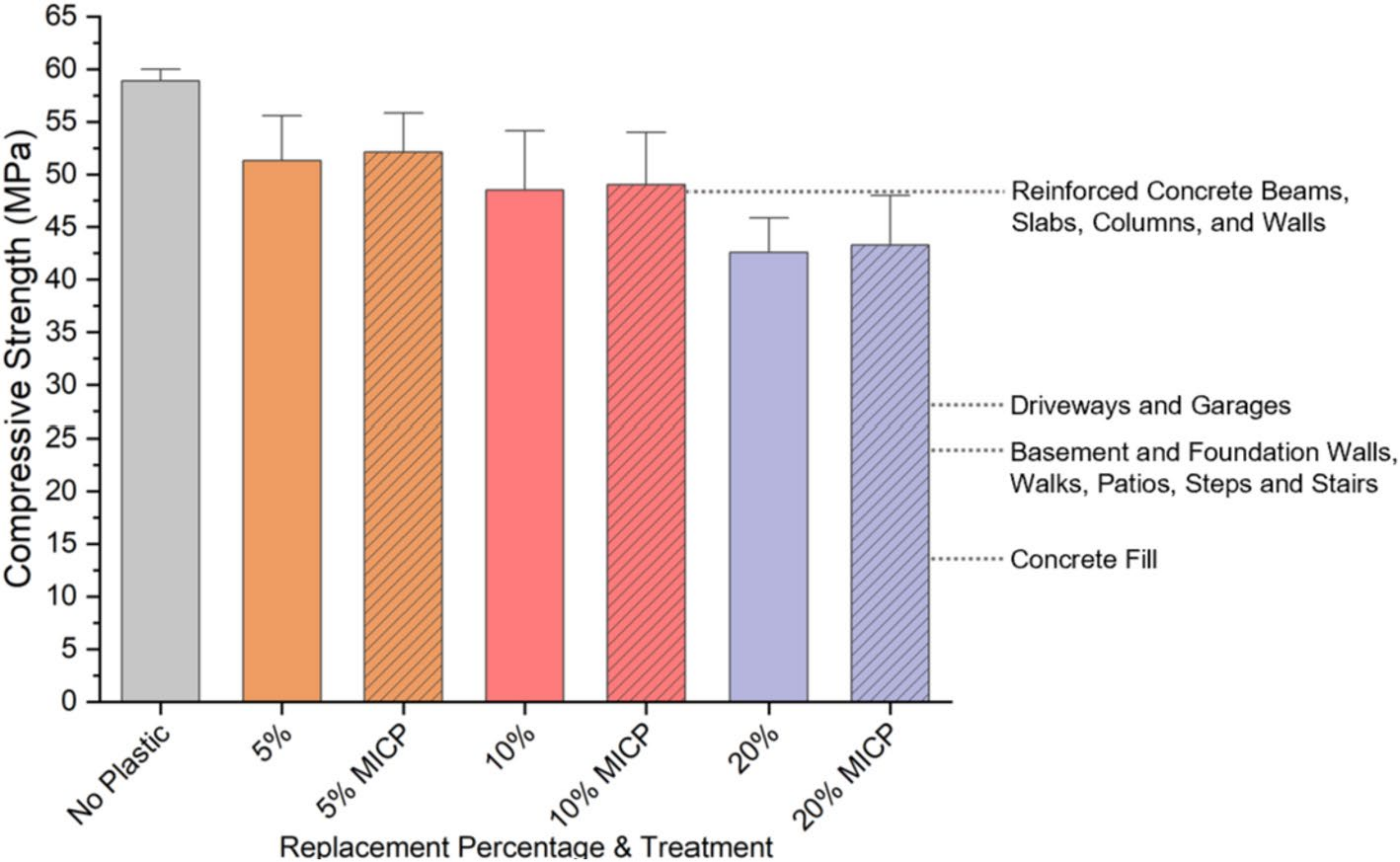
Compressive strengths at replacement percentages 5–20% with waste plastic and no treatment (solid) and MICP-treated waste plastic (striped). Strengths shown are averages across HDPE, PVC, LDPE1, LDPE2 at the given replacement percentage. The bar heights represent the mean, and the error bars indicate one standard deviation. The general compressive strengths shown for each type or location of concrete construction are the required strengths according to the International Code Council Concrete Manual: Based on the 2015 IBC^®^ and ACI 318–14. However, the strengths required for these applications may vary by design and location.

Our results demonstrate that even at a 20% replacement with untreated waste plastics (HDPE, PVC, LDPE1, and LDPE2), the resulting mortar showed promising compressive strengths, adequate for several applications, shown in Fig. 6^39^.

### Mortar strength is reduced by oil unless treated with biomineralization or washing

**Figure 7:**
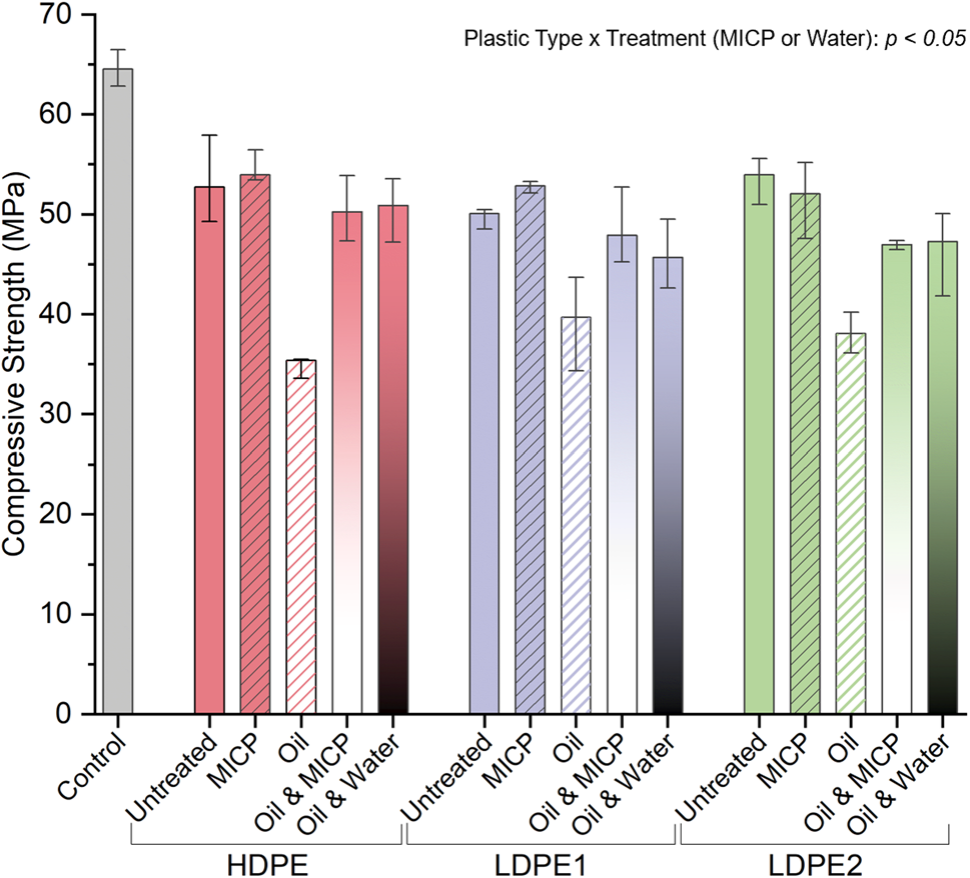
Compressive strengths at 10% volume replacement with waste plastic types HDPE (red), LDPE1 (purple), and LDPE2 (green). Each type of plastic was subjected to five treatments: no treatment (solid), MICP-treated plastic (solid and striped), oil-coated plastic (white and striped), oil-coated and MICP-treated plastic (white to solid), oil-coated and washed with water plastic (black to solid). The bars represent the mean values, and the error bars indicate one standard deviation.

To examine whether treating oil-coated plastics with MICP or washing will rescue P-FM strength, compressive tests were carried out at 10% volume replacement (Fig. 7). There was a significant interaction (*p* < 0.05) between plastic type and treatment on the compressive strength of the cylinders. Post hoc testing shows the addition of biomineral or washing of oil-coated HDPE exhibited the greatest strengths for all plastic types coated in oil. For each plastic type (HDPE, LDPE1, LDPE2) the MICP treatment or washing the oil-coated plastic with water showed a similar increase in compressive strength (*p* < 0.05). On average, biomineralization and washing of the oily plastics increased the compressive strength by 28.28% (48.37 MPa) and 27.14% (47.94 MPa), respectively, when compared to cylinders containing untreated, oil-coated plastics (37.71 MPa).

## Discussion

The objective of this research was to increase the quantity of clean and contaminated waste plastic that can be added to P-FM while maintaining adequate composite strength for common non-structural applications where more sustainable alternatives to traditional cementitious materials are desired. Increasing amounts of plastic as a replacement for aggregate has been shown to substantially decrease compressive strength^14–16,19–23,40–42^. While limited work shows that small volume replacements of cement with plastic also decrease strength, the specific relationship between larger replacement volumes, which are desirable for sustainability impacts, and compressive strength is not known^10,26^. These data are important for designing cementitious composites when strength reduction is acceptable and inclusion of greater volumes of waste plastic is the goal.

First, the impact of cement substitution with waste plastic (chipped and granular) at 5%, 10%, and 20% replacement volumes on compressive strength was assessed. As expected^14,19,26,40–44^, the 28-day compressive strength results indicated that there was an inverse relationship between the amount of plastic added and mortar strength. Despite the differences in plastic geometry and mix composition, the compressive strengths at a 5% replacement reported in the present study compare well with the strengths of mortar containing 5% untreated plastics (PVC, LDPE, PP) reported by Kane et al. At a 20% replacement with untreated waste plastics, the resulting mortar showed compressive strengths adequate for applications such as sidewalks, foundation walls, driveways, and garages (Fig. 6)^39^. In our study, the mean compressive strength of mortar prepared with a 20% cement volume replacement with plastic decreased by 27.67%, relative to plastic-free mortar. Previous studies report a loss in strength from 28.71 to 47.67% at a 20% coarse aggregate replacement with plastics in cementitious composites^19–23^. Together, these data suggest that there is potential for the sustainability of cementitious composites to improve by replacing cement, as opposed to coarse aggregate, with waste plastics.

Adding waste plastic to commonly used applications could translate to large sustainability benefits. For example, sidewalks account for nearly 7.2% (6 km^2^) of the total developed area in Barcelona, Spain as of 2006. Every year in Barcelona, 130,000 m^2^ of the sidewalk area is dug up and replaced, requiring over 14,000 metric tons of cement^45^. If 20% of the cement used to repair the sidewalks was replaced with waste plastic, at least 2,797 metric tons of cement would be saved annually, which would therefore reduce carbon dioxide emissions by around 20% while saving 913 metric tons (assuming a density of 1.028 g/cm^3^) of plastic from entering landfills^46^. This plastic waste reuse would represent a significant portion of the currently non-recoverable plastic waste generated in Barcelona^47^. This simple example demonstrates the potential for large-scale impact for utilizing large volumes of waste plastics in common non-structural applications.

The choice of waste plastic type influenced P-FM strength. The highest compressive strengths were found for LDPE2 and PVC. The differences in compressive strength may relate to different bond strengths between the plastics and the cement mortar. Espinal et al. found that the bond strength was influenced by the type of plastic and more specifically the surface energies of the plastic opposed to surface roughness or fiber tensile strength. PVC was found to have high bond strength (Espinal et al.^48^), which is in alignment with the high compressive strength of PVC-containing mortar of the current study. LDPE studied by Espinal et al. did not have high bond strength, which agrees with the compressive strength found for mortar made with chipped LDPE1 in the current study. By contrast, P-FM made with the granular LDPE2 had high compressive strength, likely signifying a relationship between the geometry of the plastic and the resulting mortar strength. Past studies identified a relationship between fiber shape or size and mechanical strength. Kim et al.^49^ found that the geometry of recycled PET fibers influenced the mechanical bond strength due to the difference in surface energy of the fibers and friction resistance during pullout. Furthermore, Pereira-de-Oliveira et al.^50^ reported that the compressive strength of cement mortar decreases when the acrylic fibers added to the mixture increase in length.

Contrary to the hypothesis of this study, a coating of MICP treatment on the plastics did not significantly increase mortar strength. These findings disagree with work by Kane et al.^21^, which showed that at a 5% weight replacement of cement paste, MICP treatment significantly increased compressive strength for PET chips and PVC short fibers. Factors that may have influenced differences in MICP effectiveness include plastic size and geometry. In previous studies, short plastic fibers have been shown to enhance mechanical properties^10–13^, while plastic as replacement for aggregate or cement demonstrates an opposite effect^19,26,40–44^. The finding that there is relatively more vaterite produced in the presence of oil is notable. Vaterite is a metastable, lower density phase of calcium carbonate and has the potential to transform into calcite. These different polymorphs may vary in their participation in cement hydration or the development of bond strength.

Other studies observed a slight increase in flexural strength through the use of MICP treatment^27,28^. However, the current study agrees with findings from several studies. Hao et al. reported that MICP treatment of PP fibers at a 1% by volume replacement did not significantly affect the compressive strength. Espinal et al.^48^ found that MICP treatment did not improve the interfacial bond strengths, frictional bond strengths, chemical bond energies, or work to pullout measures. Characteristics of the MICP coating may impact whether this treatment improves the material properties. Hao et al.^27^ reported that PP with an average coating of 0.094 g CaCO_3_/g plastic resulted in an increased fiber pull-out strength, thus improving the interfacial bonding between the plastic and the cement matrix. This fiber pullout strength was higher at a MICP coating of 0.094 g CaCO_3_/g plastic compared to coatings of 0.026 CaCO_3_/g plastic and 0.374 g CaCO_3_/g plastic^27^. However, the current study found that the average precipitate on the plastics ranged from 0.091 g CaCO_3_/g plastic for PET to 0.113 g CaCO_3_/g PVC, which are similar to the coatings achieved by Hao and co-authors. Further investigation of the exact mechanisms by which CaCO_3_ coating impacts the strength of cementitious materials is needed to determine optimal biomineral coating parameters to improve mortar strength.

Whether the strength of P-FM made using oil-contaminated plastic could by rescued through MICP treatment was also investigated. As expected, oil contamination dramatically decreased the mortar strength (e.g., the strength at 10% replacement with untreated, oil coated plastic decreased by 22.06% compared to mortar containing 10% clean plastic). MICP treatment was successful in coating the oily plastic and did improve the strength of mortar, but these benefits were not different than simply washing the oily plastic with water.

These results suggest that at the volume replacement studied (10%), it is more beneficial to simply rinse the contaminated plastic as opposed to biomineralization treatment, since microbial biomineralization requires the production of media, appropriate sterilization, and the use of chemicals (urea and calcium). Contaminated plastics may also be directly added to the mortar mixture without MICP treatment for lower-strength applications (average of 37 MPa at 10% cement replacement).

There were several limitations present in this study. Plastic filaments of uniform diameter were used to characterize, image, and weigh the biomineralized fibers opposed to chipped waste plastic because of their uniform geometry. While vegetable oil was used to mimic contaminated waste plastics, a coating of food or other oils may produce differing results than those found in this study. Additional testing is required to assess the sustainability of the mortar and to determine how MICP treatment affects other important properties of these composites such as flexural strength and durability.

## Conclusion

Experimental data indicates that P-FM manufactured with a 20% replacement of untreated plastics produced compressive strengths acceptable for several applications such as driveways, foundation walls and slabs, and concrete fill. A key finding of this study was that MICP coating on several types of common waste plastic does not significantly improve the compressive strength at replacement volumes of 5–20%. MICP was able to rescue the strength of mortar made from oil-contaminated plastic, but the benefit to strength was not greater than for simply rinsing the plastic. Together, these findings suggest that substituting a portion of the cement in mortar with clean and contaminated plastics can reduce the amount of plastic entering landfills and lessen the amount of cement needed for construction, thus potentially reducing greenhouse gas emissions.

## Supplementary Information

Supplementary Information.

## Data Availability

The data that support the findings of this study are available from the corresponding author upon reasonable request.
