# Supplementary material for: The impacts of biomineralization and oil contamination on the compressive strength of waste plastic-filled mortar
Source: Sci Rep. 2022 Dec 13;12:21547. doi: 10.1038/s41598-022-25951-3 (PMC9747956; doi:10.1038/s41598-022-25951-3)
Supplement: Supplementary file 1 — Supplementary Information. [file 41598_2022_25951_MOESM1_ESM.pdf]

Supplementary Materials: The impacts of biomineralization and oil contamination on the compressive strength of waste plastic-filled mortar

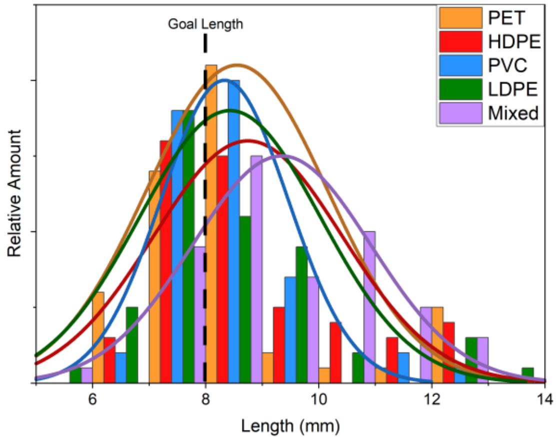

Figure S1. Distribution of fiber lengths tested in this study with a goal length of 8 mm. Filament was cut using a paper cutter fitted with a 3D-printed cutting jig. 50 fibers of each plastic type were selected randomly and measured with calipers.

Table S1. Density determined for all printer filament plastic types. Density of the plastic was calculated by recording the mass and determining the volume using caliper measurements. The resulting density was found by averaging a total of 30 plastic fibers for each plastic type.

**Plastic Type**	**Density (g/cm^3)**
PET	1.178
HDPE	0.913
PVC	1.292
LDPE	0.919
Mixed	1.027

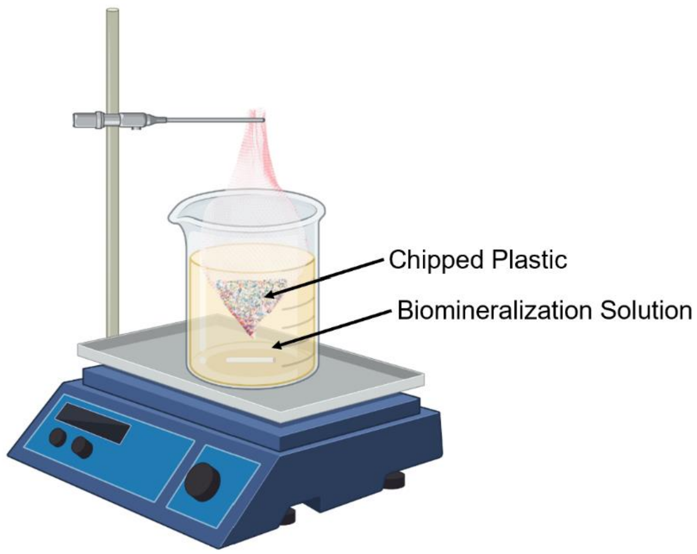

Figure S2. Biomineralization setup for precipitating calcium carbonate mineral on chipped plastic

Table S2. Mortar mix design for all plastic types at 5%, 10%, and 20% volume replacements using plastic densities shown in Table 1.

Percent Replacement	Plastic Type	Components	Total (g) (n=5)
5%	HDPE	Cement	1237.93
		Sand	2513.27
		Water	578.05
		Plastic	18.71
	PVC	Cement	1227.28
		Sand	2513.27
		Water	578.05
		Plastic	29.36
	LDPE1	Cement	1238.13
		Sand	2513.27
		Water	578.05
		Plastic	18.51
	LDPE2	Cement	1241.15
		Sand	2513.27
		Water	578.05
		Plastic	15.49

10%	HDPE	Cement	1219.22
		Sand	2513.27
		Water	578.05
		Plastic	37.41
	PVC	Cement	1197.91
		Sand	2513.27
		Water	578.05
		Plastic	58.72
	LDPE1	Cement	1219.62
		Sand	2513.27
		Water	578.05
		Plastic	37.02
	LDPE2	Cement	1225.66
		Sand	2513.27
		Water	578.05
		Plastic	61.95
20%	HDPE	Cement	1181.81
		Sand	2513.27
		Water	578.05
		Plastic	74.82
	PVC	Cement	1139.19
		Sand	2513.27
		Water	578.05
		Plastic	117.45
	LDPE1	Cement	1182.59
		Sand	2513.27
		Water	578.05
		Plastic	74.04
	LDPE2	Cement	1194.69
		Sand	2513.27
		Water	578.05
		Plastic	61.95

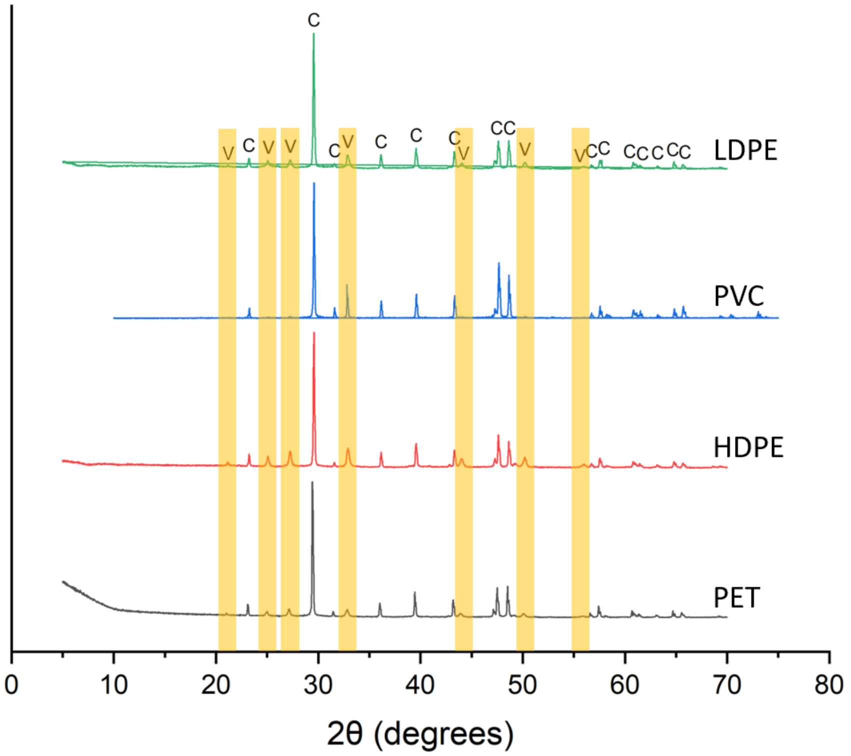

Figure S3. Vaterite is still present on oil-coated and biomineralized fibers after 145 ± 5 days of drying. Vaterite peaks are highlighted.

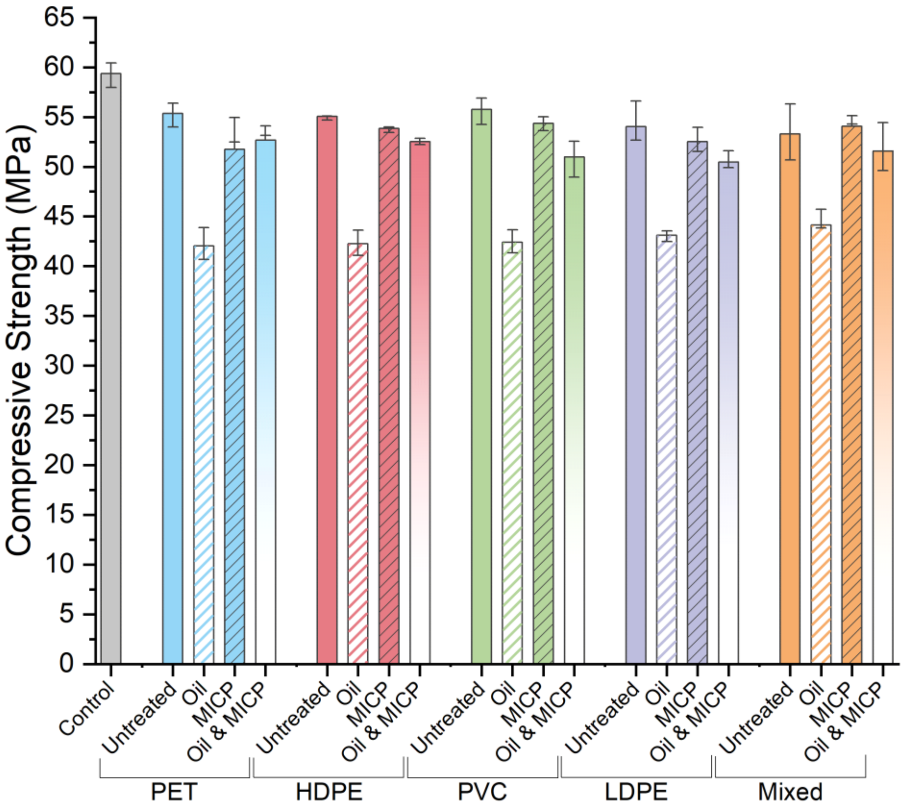

Figure S4. Preliminary experiment showing compressive strengths at 5% volume replacement with 3D printer filament (1) plastic and no treatment, (2) oil-coated plastic, (3) MICP-treated plastic, (4) oil coated, MICP-treated plastic. The bars represent the mean values, and the error bars indicate one standard deviation. Biomineralization of plastics resulted in similar strength as untreated plastics.

Table S3. Tukey post hoc results and groupings of precipitate mass and compressive strengths. Groupings of different letters signify statistically different mean values. Post hoc tests were only conducted for significant main effects and interactions.

Mass of Calcium Carbonate Precipitate Tukey Pairwise Comparisons: Plastic Type*Oil (y/n) Interaction (g)

Plastic*Oil (y/n)	N	Mean	Grouping			
LDPE y	3	16.0667	A			
PVC n	3	13.7000		B		
LDPE n	3	13.5000		B		
HDPE y	3	13.0333		B	C	
HDPE n	3	12.6667		B	C	D

PVC y	3	12.6667		B	C	D	
PET n	3	11.0333			C	D	
PET y	3	10.5000				D	

Compressive Strengths at Various Replacement Volumes Tukey Pairwise Comparisons: Plastic Type*Percentage (MPa)

**Plastic*Percentage**	**N**	**Mean**	**Grouping**				
LDPE2 5%	10	53.2213	A				
LDPE1 5%	10	51.8772	A	B			
PVC 5%	10	51.5902	A	B			
LDPE2 10%	10	50.5012		B			
PVC 10%	10	50.3482		B			
HDPE 5%	10	50.1092		B			
HDPE 10%	10	47.1483			C		
LDPE1 10%	10	47.0334			C	D	
PVC 20%	10	45.1685				D	
LDPE1 20%	10	42.6273					E
LDPE2 20%	10	42.4575					E
HDPE 20%	10	41.71981					E

Compressive Strengths at 10% Replacement with Different Treatments Tukey Pairwise Comparisons: Plastic Type*Treatment (MPa)

**Plastic*Treatment**	**N**	**Mean**	**Grouping**			
HDPE No oil, MICP, No water	5	54.0087	A			
LDPE1 No oil, MICP, No water	5	53.5532	A	B		
LDPE2 No oil, MICP, No water	5	53.1711	A	B		
HDPE No oil, No MICP, No water	5	52.9027	A	B		
LDPE2 No oil, No MICP, No water	5	52.8802	A	B		
HDPE Oil, No MICP, Water	5	50.8947	A	B	C	
HDPE Oil, MICP, No water	5	50.2544		B	C	D
LDPE1 No oil, No MICP, No water	5	50.0748		B	C	D

LDPE1 Oil, MICP, No water	5	47.8976	C	D	E		
LDPE2 Oil, No MICP, Water	5	47.2583		D	E		
LDPE2, Oil, MICP, No water	5	46.9637		D	E		
LDPE1 Oil, No MICP, Water	5	45.6700			E		
LDPE1 Oil, No MICP, No water	5	39.6759				F	
LDPE2 Oil, No MICP, No water	5	38.0632				F	G
HDPE Oil, No MICP, No water	5	35.3832					G
